# Multilevel risk and protective factors for self‐harm, suicidal ideation and suicide attempt in adolescents

**DOI:** 10.1111/jcpp.70024

**Published:** 2025-08-04

**Authors:** Alison L. Calear, Philip J. Batterham, Aliza Werner‐Seidler, Kate Maston, Michelle Torok, Bridianne O'Dea, Mark E. Larsen, Helen Christensen

**Affiliations:** ^1^ Centre for Mental Health Research The Australian National University Canberra ACT Australia; ^2^ Black Dog Institute University of New South Wales Sydney NSW Australia; ^3^ Flinders University Institute for Mental Health and Wellbeing Flinders University Adelaide SA Australia; ^4^ Centre for Big Data Research in Health University of New South Wales Sydney NSW Australia; ^5^ Discipline of Psychiatry and Mental Health University of New South Wales Sydney NSW Australia

**Keywords:** Suicide, self‐harm, adolescent, risk factors, protective factors

## Abstract

**Background:**

Better characterising risk and protective factors for suicidal distress and self‐harm in adolescents may facilitate better targeting of interventions that address underlying vulnerabilities. However, few previous longitudinal studies have: (1) sufficient power to identify key risk and protective factors, (2) limited representativeness to the community and (3) accounted for multilevel factors (individual, family, community). This study aimed to assess prevalence and identify risk and protective factors for self‐harm, suicidal ideation and suicide attempts in a large cohort of Australian adolescents.

**Methods:**

Data from 4,122 adolescents from 134 Australian schools were collected as part of the Future Proofing Study, a prospective cohort study of adolescent mental health and wellbeing. Generalised linear mixed models were used to assess the effect of baseline mental health, lifestyle, social and school‐level factors on self‐harm, suicidal ideation and suicide attempt at 12‐month follow‐up.

**Results:**

At 12‐month follow‐up, 17.7% of adolescents reported self‐harming behaviour, 18.6% reported suicidal ideation and 3.0% reported a suicide attempt. In addition to mental health history, female and gender‐diverse identities, LGBTQA+ identity and greater levels of prosocial behaviour were significantly associated with self‐harm and suicidal ideation. Peer problems were associated with suicidal ideation and suicide attempt.

**Conclusions:**

Rates of suicidal distress and self‐harm remain high in Australian adolescents. Reducing symptoms of depression, improving peer relationships, mitigating online bullying and providing social support for families may be suitable targets for future prevention and early intervention programs.

Youth suicide is a significant public health challenge. Globally, suicide accounts for 8.5% of all deaths in adolescents and young adults (Cha et al., 2018) and is estimated to be the second leading cause of death in this population (Glenn et al., 2020; World Health Organization, 2014). A recent systematic review and meta‐analysis of suicide phenomena in young people worldwide estimated the proportion of adolescents reporting a suicide attempt or suicidal thoughts in the past 12 months as 5.2% and 15.1%, respectively (Van Meter, Knowles, & Mintz, 2023). The prevalence of suicidal ideation rapidly increases between the ages of 12 and 17, and over a third of adolescents who experience suicidal ideation go on to make a suicide attempt (Nock et al., 2013). These arresting figures are associated with significant fiscal and societal costs, including emotional and psychosocial difficulties, medical care and lost productivity (Kinchin & Doran, 2018).

Self‐harm, defined as intentionally causing pain or damage to one's own body with or without suicidal intent (Moran et al., 2024), is a significant risk factor for suicide (Wilkinson et al., 2022). There is evidence of increasing rates of self‐harm among children and adolescents in many high‐income countries (Gillies et al., 2018; Sara et al., 2023; Tørmoen, Myhre, Walby, Grøholt, & Rossow, 2020). A meta‐analysis of nonsuicidal self‐injury among children and adolescents reported the mean lifetime prevalence of self‐harm in children and adolescents to be 22.1% (Lim et al., 2019). Relatedly, a report by the Australian Institute of Health and Welfare (2023) recently found that young people have the highest rates of hospitalisation for intentional self‐harm in Australia and rates are increasing for young females. With such high rates of self‐harm, suicidal ideation and suicide attempts in young people, it is imperative that we gain a better understanding of the risk and protective factors associated with these outcomes to enable early identification of at‐risk young people, as well as the development of prevention and early intervention programs targeting these factors to reduce risk.

A recent umbrella review by Richardson et al. (2024) synthesised 20 years of research on the risk and protective factors associated with self‐harm and suicide in adolescents. In this review and others (van Geel, Goemans, & Vedder, 2015), both victims and perpetrators of bullying (or peer victimisation) were identified as being at increased risk of suicidal ideation, suicide attempts and self‐harm, with cyberbullying found to be associated with even higher risks than face‐to‐face bullying (Richardson et al., 2024; Wasserman, Carli, Iosue, Javed, & Herrman, 2021). A strong relationship between poor peer relationships and self‐harm, suicidal ideation and attempts is also evident in the literature and encapsulates constructs such as loneliness, low friendship support, peer‐related stressors and conflict (De Luca, Giletta, Menesini, & Prinstein, 2022; Evans, Hawton, & Rodham, 2004). Sleep disturbance and antidepressant use were also associated with self‐harm and suicidality (Richardson et al., 2024). Vulnerable youth populations included females (Cha et al., 2018; Richardson et al., 2024; Valencia‐Agudo, Burcher, Ezpeleta, & Kramer, 2018), young people identifying as lesbian, gay, bisexual, transgender, queer (or questioning; LGBTQA+) (Richardson et al., 2024; Surace et al., 2021) and those with specific mental health disorders, such as depression, anxiety, conduct problems and attention deficit hyperactivity disorder (ADHD) (Fang et al., 2024; Garas & Balazs, 2020; Richardson et al., 2024; Valencia‐Agudo et al., 2018; Wasserman et al., 2021), or previous suicidality and self‐harm (Richardson et al., 2024; Valencia‐Agudo et al., 2018).

Several studies have also assessed the association between social media and internet use and suicidal ideation, suicide attempts and self‐harm, with reviews reporting limited evidence of an association between the frequency of social media use and self‐harm and suicide (Cha et al., 2018; Nesi et al., 2021). The assessment of protective factors has been less common, although school connectedness and parental support have consistently been shown in cross‐sectional and longitudinal studies to be associated with lower risks of suicidal ideation, suicide attempts and self‐harm across multiple reviews (Evans et al., 2004; Marraccini & Brier, 2017; Richardson et al., 2024; Valencia‐Agudo et al., 2018; Wasserman et al., 2021).

While a diverse range of studies has been conducted in this field, most have assessed a small number of factors, focused only on self‐harm, suicidal ideation or suicide attempts or have been limited to the assessment of cross‐sectional associations (Cha et al., 2018). To address these limitations, the current study aims to identify (1) rates of self‐harm, suicidal ideation and suicide attempts in a large, contemporary cohort study of Australian adolescents and (2) assess, longitudinally, the association between a range of mental health, social, lifestyle and school‐level factors and self‐harm, suicidal ideation and suicide attempts.

## Methods

### Design, participants and procedure

This paper utilised baseline and 12‐month follow‐up data from the Future Proofing Study, a prospective cohort study of adolescent mental health and wellbeing that included an embedded cluster randomised controlled trial to assess the effectiveness of a 6‐week cognitive behavioural therapy‐based intervention to prevent depression compared with a no intervention control condition (ACTRN12619000855123). The Future Proofing Study received ethics approval from the University of New South Wales Human Research Ethics Committee (HC180836), the NSW Government State Education Research Applications Process Approval (SERAP 2019201) and relevant Catholic Schools Dioceses across Australia.

The Future Proofing Study was conducted in 134 secondary schools located across Australia, including government, independent and Catholic schools. All government and independent secondary schools located in New South Wales (NSW) and eligible NSW Catholic secondary schools were invited to participate. Independent schools in other Australian capital cities were also invited to participate. The preponderance of schools from NSW was by design, to minimise logistical barriers associated with in‐school data collection conducted by the research team. In total, 1,036 schools were invited to participate. Reasons for nonparticipation included lack of interest, limited time or pressures due to the pandemic (Werner‐Seidler et al., 2025). Overall, participant characteristics indicate that the Future Proofing Study cohort is broadly representative of the Australian adolescent population, including in relation to gender, remoteness, Aboriginal and/or Torres Strait Islander identity, LGBTQA+ identity and clinical profiles (Werner‐Seidler et al., 2023).

Baseline data collection took place across three separate Year 8 cohorts (students aged 13–14 years): the 2019 cohort between August and September 2019; the 2020 cohort between August and November 2020; and the 2021 cohort between April 2021 and March 2022. Parents/guardians and students provided active informed consent and assent, respectively, for students to participate in the study. Participants completed the baseline and 12‐month follow‐up assessments at school in sessions facilitated by the research team. Students accessed the assessments via a secure website, with each taking approximately 45 min to complete. Full trial details are provided in the protocol and cohort papers (Werner‐Seidler et al., 2020, 2023).

Given the null effect of the prevention program on depressive symptoms and secondary outcomes of psychological distress, anxiety and insomnia (Standardised mean difference = 0.00–0.16; *p*‐values .12–.99) (Werner‐Seidler et al., 2025), participants in both the intervention and control conditions were eligible for inclusion in the current study. At baseline, 5,720 students fully completed an assessment. A total of 4,122 (72%) of these students also completed a 12‐month assessment and were included in the analysis. Missing data at 12‐month follow‐up were not missing completely at random (MCAR; Little's test *χ* ^2^ = 1,226.4, *p* < .001), although MCAR was not an assumption of the models used. Based on univariate analyses, attrition from baseline to 12‐month follow‐up was significantly higher among students with poorer mental health, including greater levels of self‐harm, suicidal ideation, suicide attempt, depression symptoms, conduct problems, hyperactivity, peer problems and insomnia (*p* < .05 for all). Participants with poorer school climate, less school connectedness, less prosocial behaviour, more bullying or cyberbullying, male gender, government schools or with a mental health diagnosis (*p* < .05 for all) also had significantly higher attrition. It is noted that even small differences in attrition (*d* < 0.1) were significant in this large sample. Presence of suicidal ideation at baseline marginally increased attrition from 23% to 26%. Intervention condition and anxiety symptoms were not significantly associated with attrition.

### Measures

#### Self‐harm

A single item from the Self‐Harm Questionnaire (SHQ; Ougrin & Boege, 2013) was used to assess past episodes of self‐harm. Participants responded to the item ‘Have you ever actually harmed yourself on purpose? For example, have you ever cut yourself or taken an overdose and it was not an accident?’ on a 4‐point scale of *No*, *Yes, once*, *Yes, two, three or four times* and *Yes, five or more times*. For analysis, responses to this item were dichotomised to indicate the presence of self‐harming behaviour (Yes/No).

#### Suicidal ideation

The 5‐item Suicidal Ideation Attributes Scale (SIDAS; van Spijker et al., 2014) was used to measure suicidal ideation severity in the past month. Items in this measure were rated on a scale from 0 to 10 (with differing scale endpoints) and assessed the frequency and controllability of suicidal thoughts, distress and impairment related to suicidal thoughts, and closeness to suicide attempt. Total SIDAS scores were calculated as the sum of the five items, with the controllability item reverse scored. Total scale scores on the SIDAS range from 0 to 50, with higher scores indicative of greater suicidal ideation severity. The SIDAS demonstrated good internal consistency in the current study (Cronbach's *α* = .92).

#### Suicide attempt

A single item from the Youth Risk Behaviour Survey (YRBS; Centers for Disease Control and Prevention, 2015) was used in the current study to assess the presence of suicidal behaviour (suicide attempt(s)) in the past 12 months. Participants were instructed to report the number of suicide attempts they had made in the past 12 months. This item was subsequently dichotomised into ‘no attempts’ (0) or ‘one or more attempts’ (1) and used in the current study to assess past suicide attempts. Previous studies have shown that the YRBS suicidality items demonstrate both reliability (Brener et al., 2002) and good convergent and divergent validity in a secondary school sample (May & Klonsky, 2011).

#### Mental health symptoms

The 9‐item Patient Health Questionnaire for Adolescents (PHQ‐A; Johnson, Harris, Spitzer, & Williams, 2002) was used to assess symptoms of depression (*α* = .88), while the 8‐item Children's Anxiety Scale–Short Form (CAS‐8; Spence et al., 2014) was used to assess anxiety (*α* = .90). The 5‐item ‘conduct problems’ (*α* = .63) and ‘hyperactivity/inattention’ (*α* = .78) sub‐scales of the self‐rated Strengths and Difficulties Questionnaire (SDQ; Goodman, 1997) were administered to assess externalising symptoms.

#### Social factors

The 5‐item ‘family member’ sub‐scale (*α* = .80) of the Schuster Social Support Scale (SSS; Schuster, Kessler, & Aseltine Jr., 1990) was used to measure positive and negative interactions with family members, while the 5‐item ‘peer relationship problems’ (total score *α* = .56) and ‘prosocial behaviour’ (*α* = .66) sub‐scales of the self‐rated SDQ (Goodman, 1997) were administered to assess peer relationships. Single items, derived from previous school‐based trials (Calear et al., 2016), were used to assess how frequently participants had been bullied or cyber‐bullied in the past 12 months (not at all, a few times, once a month, once a week, most days).

#### Lifestyle factors

The 7‐item Insomnia Severity Index (ISI; Bastien, Vallières, & Morin, 2001) was used to assess insomnia symptoms over the previous 2 weeks (*α* = .86). A single item was used to assess the number of recreational screen time hours per day (0–1, 1–2, 2–3, 3–4, 4–5, 5+ hr), and an adapted version of the 7‐item Maladaptive Facebook Usage Scale (Smith, Hames, & Joiner Jr., 2013) was used to assess participant tendency to undertake negative social evaluations and social comparisons when using social media (*α* = .76). The scale was adapted to apply to all social media use, rather than focusing just on Facebook use (e.g. When I update my social media status and no one comments on it, I tend to be disappointed), with participants agreeing or disagreeing with each item.

#### School‐level factors

School sector was a single dichotomised variable reported by each school differentiating government and nongovernment (Independent and Catholic) schools, while school connectedness was assessed at the individual level using the 6‐item self‐report measure (*α* = .87) developed by the OECD Programme for International Student Development (OECD, 2004). School climate was also assessed using a 7‐item self‐report measure (Fink, Patalay, Sharpe, & Wolpert, 2018), which was measured at the individual level (*α* = .85). Socio‐educational advantage was measured using the Index of Community Socio‐Educational Advantage (ICSEA), a measure that is available from a database curated by the Australian Curriculum, Assessment and Reporting Authority. ICSEA is an index applied to all Australian schools and is calculated from parent and community data. Scores are standardised such that there is a mean of 1,000, and higher scores represent greater levels of advantage.

#### Demographic factors

A range of socio‐demographic variables was also collected via self‐report including age, gender (male/female/other gender identity), sexuality (LGBTQA+ vs. heterosexual), mental health diagnosis history, location (outer regional/inner regional/major city) and language spoken at home (English vs. other).

#### Analysis

Participant characteristics and rates of self‐harm, suicidal ideation and attempts at baseline and 12‐month follow‐up were reported using descriptive statistics. Generalised linear mixed models (including a logit link function for binary outcomes) were used to assess the association between baseline mental health, lifestyle, social and school‐level factors and self‐harm, suicidal ideation and suicide attempt at 12‐month follow‐up. A random intercept for school was included in the models to account for potential clustering by school. A hierarchical approach was taken, with baseline mental health, lifestyle, social‐ and school‐level factors entered in Model 1 and baseline self‐harm, suicidal ideation and/or suicide attempt entered in Model 2 where appropriate. Demographic factors and trial condition were also included in the models in Step 1 to assess and control for their effects on the outcome measures. All the risk and protective factors were included in a single step as the study was sufficiently powered to do so; there was little evidence of multicollinearity, there were no a priori expectations about how to enter/cluster factors together or about which factors might be of greater interest, and interaction terms were not tested to explore specific mediators. All analyses were conducted in SPSS v27.

## Results

### Participant characteristics

Table 1 presents participant characteristics. Overall, 4,122 participants were included in the analysis. Ninety‐eight percent of the sample was aged between 12 and 14 years (*M* = 13.92, *SD* = 0.58) at baseline. Just over half of participants (51.2%) identified as female, while 43.9% identified as male and 4.9% endorsed other gender identities. Over a quarter of participants (27.6%) reported a nonheterosexual identity. Most of the sample spoke English as their first language (93.4%) were located in a major city (76.2%) and attended a government school (52.6%).

**Table 1:** Participant characteristics and study measures (*N* = 4,122)

	*n*	%
Gender		
Male	1,808	43.9%
Female	2,111	51.2%
Other gender identity	203	4.9%
Government school	2,170	52.6%
Location		
Inner regional	898	21.8%
Major city	3,140	76.2%
Outer regional	84	2.0%
Language: Other vs. English	270	6.6%
Nonheterosexual identity	1,137	27.6%
Screen time ≥3 hr vs. <3 hr	2,667	64.7%
Intervention condition	2,099	50.9%
MH diagnosis	641	15.6%
Self‐harm at baseline	869	21.1%
Self‐harm at 12 months	730	17.7%
Suicide attempt at baseline	160	3.9%
Suicide attempt at 12 months	123	3.0%

	Mean	*SD*
Age	13.92	0.58
SIDAS at baseline	2.95	7.34
SIDAS at 12 months	2.76	7.96
PHQ‐A depression	7.27	6.06
CAS‐8 anxiety	8.33	5.40
SDQ conduct problems	2.05	1.86
SDQ hyperactivity	4.66	2.62
SDQ peer problems	2.26	1.80
SDQ prosocial	7.22	1.97
ISI insomnia symptoms	7.03	5.44
Positive family interactions	2.56	0.65
Negative family interactions	1.29	0.84
Bullying frequency	1.57	0.97
Cyberbullying frequency	1.26	0.65
Maladaptive social media use	17.49	7.20
School connectedness	17.79	3.57
School climate	8.78	3.21

Other gender identities include nonbinary, other specified identities and prefer not to answer responses; ‘nonheterosexual’ identity includes gay, lesbian, bisexual, asexual, transgender, queer and other specified sexual orientations other than heterosexual; ‘other’ language includes any language spoken instead of or in addition to English at home. CAS‐8, Children's Anxiety Scale–Short form; ISI, Insomnia Severity Index; MH, mental health; PHQ‐A, Patient Health Questionnaire for Adolescents; SDQ, Strengths and Difficulties Questionnaire; SIDAS, Suicidal Ideation Attributes Scale.

At baseline, 21.1% of participants reported self‐harming behaviour. This figure dropped to 17.7% at 12‐month follow‐up. Reporting of self‐harm was inconsistent across the two assessments, with 405 (9.8%) participants who reported lifetime self‐harm at baseline not reporting it at 12 months. In total, 1,135 (27.5%) participants reported self‐harm at one or both time points. Mean suicidal ideation (SIDAS) scores also decreased from baseline to 12‐month follow‐up, reducing from 2.95 (*SD* = 7.34) to 2.76 (*SD* = 7.96). To ascertain rates of suicidal ideation at baseline and 12‐month follow‐up, the proportion of participants reporting nonzero SIDAS scores (indicating the presence of suicidal ideation) was 23.9% at baseline and 18.6% at 12‐month follow‐up. Finally, 3.9% of participants reported a suicide attempt in the previous 12 months at baseline, while 3.0% reported a suicide attempt at the 12‐month follow‐up.

### Predictors of self‐harm

Table 2 presents the results of the binary mixed models assessing the association between baseline mental health, lifestyle, social‐ and school‐level factors on self‐harm at 12‐month follow‐up. In the final model, 12‐month self‐harm was significantly associated with gender, having a mental health diagnosis, attendance at a government school, identifying as LGBTQA+, higher baseline SDQ prosocial and suicidal ideation scores, and presence of self‐harming behaviour at baseline. Specifically, there were 43% lower odds of self‐harm at 12‐month follow‐up for male participants and 52% higher odds for nonbinary participants compared with female participants, 71% higher odds of self‐harm among those with a previous mental health diagnosis, 57% higher odds for those identifying as LGBTQA+, 32% higher odds among participants attending a government school, 6% higher odds per one unit increase in prosocial scores, 1% higher odds per one unit increase in suicidal ideation scores and 6.6 times the odds for people reporting self‐harm at baseline. Higher baseline depression and positive family interaction scores were significantly associated with self‐harm at 12 months in Model 1 but were not significant in the final model with the addition of baseline suicidal ideation and self‐harm. All other factors were not found to be significantly associated with self‐harm in either model.

**Table 2:** Mixed effects model of self‐harm at 12 months, accounting for clustering by school

Parameter	Model 1: All predictors except SH/SI					Model 2: All predictors + baseline SH/SI				
	*B*	*SE*	*t*	OR	*p*	*B*	*SE*	*t*	OR	*p*
Intercept	−3.72	0.89	−4.19	0.02	<.001	−4.44	0.99	−4.50	0.01	<.001
Condition (intervention vs. control)	−0.03	0.09	−0.31	0.97	.756	0.03	0.10	0.31	1.03	.759
Gender										
Male	−0.49	0.11	−4.60	0.62	**<.001**	−0.56	0.11	−4.88	0.57	**<.001**
Other gender identity	0.36	0.17	2.09	1.43	.**037**	0.42	0.19	2.16	1.52	.**031**
Female (reference)										
MH diagnosis	0.67	0.11	6.27	1.95	**<.001**	0.54	0.12	4.53	1.71	**<.001**
Government school	0.31	0.10	3.07	1.36	.**002**	0.28	0.11	2.52	1.32	.**012**
Location										
Inner regional	−0.01	0.32	−0.02	0.99	.982	0.00	0.35	0.01	1.00	.992
Major city	−0.09	0.32	−0.28	0.91	.782	−0.09	0.36	−0.25	0.91	.803
Outer regional (reference)										
Language: Other vs. English	0.11	0.17	0.65	1.12	.513	0.01	0.19	0.07	1.01	.942
LGBTQA+ vs. heterosexual	0.52	0.10	5.31	1.68	**<.001**	0.45	0.11	4.17	1.57	**<.001**
Screen time ≥3 hr vs. <3 hr	−0.06	0.10	−0.57	0.94	.570	−0.12	0.11	−1.07	0.89	.285
Socioeconomic advantage	0.00	0.00	1.07	1.00	.284	0.00	0.00	1.57	1.00	.118
PHQ‐A depression	0.08	0.01	7.07	1.09	**<.001**	0.02	0.01	1.67	1.02	.095
CAS‐8 anxiety	0.00	0.01	−0.17	1.00	.864	−0.02	0.01	−1.22	0.98	.222
SDQ conduct problems	0.05	0.03	1.82	1.05	.070	0.03	0.03	1.03	1.03	.304
SDQ hyperactivity	0.02	0.02	1.05	1.02	.295	0.04	0.02	1.50	1.04	.134
SDQ peer problems	0.05	0.03	1.72	1.06	.085	0.05	0.03	1.41	1.05	.159
SDQ prosocial	0.07	0.03	2.88	1.07	.**004**	0.06	0.03	2.10	1.06	.**036**
ISI insomnia symptoms	0.00	0.01	−0.13	1.00	.896	0.01	0.01	0.74	1.01	.462
Bullying frequency	0.01	0.05	0.21	1.01	.836	−0.01	0.05	−0.19	0.99	.851
Cyberbullying frequency	0.09	0.07	1.43	1.10	.154	0.09	0.07	1.28	1.10	.202
School connectedness	−0.02	0.02	−0.94	0.98	.346	−0.01	0.02	−0.69	0.99	.492
School climate	0.01	0.02	0.67	1.01	.504	0.01	0.02	0.44	1.01	.660
Positive family interactions	−0.21	0.07	−2.94	0.81	.**003**	−0.05	0.08	−0.61	0.95	.544
Negative family interactions	0.08	0.06	1.24	1.08	.215	0.06	0.07	0.81	1.06	.416
Maladaptive social media use	0.00	0.01	0.25	1.00	.799	0.00	0.01	−0.12	1.00	.906
Suicidal ideation (baseline)						0.01	0.01	2.23	1.01	.**026**
Self‐harm (baseline)						1.88	0.11	16.95	6.57	**<.001**

All bold values are significant at the *p* < .05 level.

CAS‐8, Children's Anxiety Scale–Short Form; *df*, degrees of freedom; ISI, Insomnia Severity Index; PHQ‐A, Patient Health Questionnaire for Adolescents; SDQ, Strengths and Difficulties Questionnaire; *SE*, standard error; SH, self‐harm; SI, suicidal ideation.

### Predictors of suicidal ideation

Table 3 presents the linear mixed models assessing the association between baseline mental health, lifestyle, social‐ and school‐level factors and suicidal ideation severity at 12‐month follow‐up. In the final model, higher suicidal ideation at 12 months was significantly associated with nonbinary and female gender, having a previous mental health diagnosis, identifying as LGBTQA+, reporting recreational screen use of <3 h per day, reporting a higher frequency of being cyber‐bullied, higher baseline depressive symptoms, peer problem and prosocial scores, and higher baseline self‐harm and suicidal ideation. Lower baseline positive family interaction scores were significantly associated with higher suicidal ideation at 12 months in Model 1 but became nonsignificant in the final model with the addition of baseline self‐harm and suicidal ideation. No other factors were found to be significantly associated with suicidal ideation in either model.

**Table 3:** Mixed effects model of suicidal ideation severity (SIDAS) at 12 months, accounting for clustering by school

Parameter	Model 1: All predictors except SH/SI					Model 2: All predictors + baseline SH/SI				
	*B*	*SE*	*df*	*t*	*p*	*B*	*SE*	*df*	*t*	*p*
Intercept	1.81	2.93	153.90	0.62	.537	1.21	2.82	168.64	0.43	.668
Condition (intervention vs. control)	0.15	0.30	66.60	0.50	.617	0.29	0.29	71.58	1.01	.316
Gender										
Male	−0.71	0.31	1018.56	−2.31	.**021**	−0.83	0.30	1065.46	−2.80	.**005**
Other gender identity	1.50	0.63	4164.67	2.36	.**018**	1.60	0.63	4083.94	2.55	.**011**
Female (reference)										
MH diagnosis	1.60	0.37	4165.76	4.33	**<.001**	0.94	0.36	4083.94	2.63	.**009**
Government school	0.40	0.35	57.75	1.15	.257	0.34	0.34	62.32	1.02	.311
Location										
Inner regional	0.14	1.00	413.96	0.14	.891	0.68	0.96	446.27	0.71	.478
Major city	0.01	1.01	372.09	0.01	.990	0.69	0.98	401.36	0.71	.480
Outer regional (reference)										
Language: Other vs. English	−0.41	0.54	3514.80	−0.77	.443	−0.65	0.52	3430.78	−1.26	.207
LGBTQA+ vs. heterosexual	1.28	0.32	4117.54	4.06	**<.001**	1.13	0.30	4046.21	3.71	**<.001**
Screen time ≥3 hr vs. <3 hr	−0.63	0.28	4153.84	−2.24	.**025**	−0.61	0.27	4075.83	−2.24	.**025**
Socioeconomic advantage	0.00	0.00	86.36	−0.52	.607	0.00	0.00	93.91	−0.61	.540
PHQ‐A depression	0.32	0.04	4165.15	8.13	**<.001**	0.13	0.04	4082.65	3.19	.**001**
CAS‐8 anxiety	0.01	0.04	4165.91	0.35	.723	−0.02	0.04	4084.00	−0.41	.679
SDQ conduct problems	0.14	0.09	4165.45	1.61	.108	0.06	0.09	4083.47	0.70	.485
SDQ hyperactivity	−0.06	0.06	4164.88	−0.85	.396	−0.01	0.06	4083.38	−0.09	.929
SDQ peer problems	0.28	0.10	4166.00	2.92	.**003**	0.27	0.09	4083.98	2.92	.**003**
SDQ prosocial	0.22	0.07	4165.96	3.00	.**003**	0.19	0.07	4084.00	2.69	.**007**
ISI insomnia symptoms	0.01	0.03	4164.31	0.34	.732	0.05	0.03	4083.14	1.40	.161
Bullying frequency	−0.17	0.16	4165.85	−1.04	.300	−0.28	0.16	4083.99	−1.80	.071
Cyberbullying frequency	0.67	0.23	4164.41	2.94	.**003**	0.59	0.22	4081.91	2.66	.**008**
School connectedness	−0.09	0.05	4159.93	−1.68	.094	−0.07	0.05	4078.32	−1.30	.195
School climate	0.02	0.05	4113.33	0.47	.642	−0.03	0.05	4042.36	−0.67	.501
Positive family interactions	−0.73	0.23	4164.78	−3.13	.**002**	−0.22	0.23	4082.55	−0.95	.342
Negative family interactions	0.35	0.19	4166.00	1.87	.061	0.27	0.18	4083.72	1.50	.135
Maladaptive social media use	0.00	0.02	4153.70	−0.26	.798	−0.01	0.02	4069.55	−0.55	.584
Suicidal ideation (baseline)						0.22	0.02	4081.21	10.28	**<.001**
Self‐harm (baseline)						1.66	0.37	4082.52	4.47	**<.001**

All bold values are significant at the *p* < .05 level.

CAS‐8, Children's Anxiety Scale–Short Form; *df*, degrees of freedom; ISI, Insomnia Severity Index.; PHQ‐A, Patient Health Questionnaire for Adolescents; SDQ, Strengths and Difficulties Questionnaire; SE, standard error; SH, self‐harm; SI, suicidal ideation; SIDAS, Suicidal Ideation Attributes Scale.

### Predictors of suicide attempt

Table 4 shows the results of the binary mixed models assessing the association between baseline factors and suicide attempt at 12‐month follow‐up. In the final model, reported suicide attempt at 12‐month follow‐up was significantly associated with having higher baseline SDQ peer problem, self‐harm and suicidal ideation scores and reporting a suicide attempt in the past 12 months at baseline. More specifically, there were 19% higher odds per one unit increase in peer problem scores, 3% higher odds per one unit increase in suicidal ideation scores, 2.2 times the odds for people reporting lifetime self‐harm and 2.8 times the odds for people reporting a suicide attempt in the previous 12 months at baseline. Previous mental health diagnosis, higher baseline depression and greater frequency of cyberbullying were found to be significantly associated with suicide attempt at 12 months in Model 1 but were not significant in the final model with the addition of baseline self‐harm, suicidal ideation and suicide attempt. All other factors were not found to be significantly associated with suicide attempt in either model.

**Table 4:** Mixed‐effects model of suicide attempt at 12 months, accounting for clustering by school

Parameter	Model 1: All predictors except SH/SI/SA					Model 2: All predictors + baseline SH/SI/SA				
	*B*	*SE*	*t*	OR	*p*	*B*	*SE*	*t*	OR	*p*
Intercept	−2.16	1.85	−1.17	0.11	.243	−2.78	2.16	−1.29	0.06	.199
Condition (intervention vs. control)	−0.14	0.18	−0.77	0.87	.441	−0.04	0.21	−0.18	0.96	.859
Gender										
Male	−0.03	0.22	−0.14	0.97	.888	−0.25	0.25	−1.00	0.78	.319
Other gender identity	0.43	0.28	1.54	1.54	.122	0.35	0.35	1.01	1.42	.311
Female (reference)										
MH diagnosis	0.64	0.19	3.33	1.90	<.**001**	0.44	0.23	1.89	1.56	.058
Government school	−0.04	0.22	−0.19	0.96	.850	0.00	0.25	0.00	1.00	.997
Location										
Inner regional	−0.05	0.54	−0.10	0.95	.924	−0.28	0.66	−0.43	0.75	.666
Major city	0.04	0.55	0.07	1.04	.944	−0.21	0.67	−0.31	0.81	.756
Outer regional (reference)										
Language: Other vs. English	0.26	0.35	0.74	1.30	.458	0.22	0.39	0.57	1.25	.569
LGBTQA+ vs. heterosexual	0.14	0.21	0.68	1.15	.495	−0.07	0.24	−0.29	0.93	.775
Screen time ≥3 hr vs. <3 hr	−0.26	0.21	−1.24	0.77	.216	−0.44	0.24	−1.85	0.64	.064
Socioeconomic advantage	0.00	0.00	−1.74	1.00	.081	0.00	0.00	−0.80	1.00	.424
PHQ‐A depression	0.07	0.02	2.88	1.07	.**004**	0.00	0.03	−0.03	1.00	.978
CAS‐8 anxiety	−0.03	0.02	−1.19	0.97	.233	−0.06	0.03	−1.90	0.95	.057
SDQ conduct problems	0.10	0.05	1.84	1.10	.066	0.06	0.06	0.98	1.06	.329
SDQ hyperactivity	0.02	0.05	0.45	1.02	.653	0.06	0.06	1.03	1.06	.306
SDQ peer problems	0.19	0.06	3.24	1.21	.**001**	0.18	0.07	2.56	1.19	.**010**
SDQ prosocial	0.02	0.05	0.50	1.02	.618	0.02	0.06	0.32	1.02	.752
ISI insomnia symptoms	0.03	0.02	1.53	1.03	.127	0.04	0.02	1.61	1.04	.107
Bullying frequency	−0.02	0.09	−0.21	0.98	.834	0.01	0.10	0.08	1.01	.933
Cyberbullying frequency	0.20	0.10	2.02	1.22	.**043**	0.16	0.12	1.35	1.18	.178
School connectedness	−0.05	0.03	−1.53	0.95	.127	−0.04	0.04	−1.06	0.96	.288
Positive family interactions	0.02	0.03	0.71	1.02	.481	0.00	0.04	0.07	1.00	.943
Negative family interactions	−0.12	0.13	−0.91	0.89	.361	−0.05	0.16	−0.34	0.95	.734
Maladaptive social media use	−0.05	0.13	−0.37	0.95	.708	−0.02	0.15	−0.13	0.98	.899
School climate	0.02	0.01	1.79	1.02	.073	0.01	0.01	0.71	1.01	.480
Suicidal ideation (baseline)						0.03	0.01	2.73	1.03	.**006**
Suicide attempt (baseline)						1.04	0.30	3.45	2.82	**<.001**
Self‐harm (baseline)						0.78	0.27	2.89	2.19	.**004**

All bold values are significant at the *p* < .05 level.

CAS‐8, Children's Anxiety Scale–Short Form; *df*, degrees of freedom; ISI, Insomnia Severity Index; PHQ‐A, Patient Health Questionnaire for Adolescents; SA, suicide attempt; SDQ, Strengths and Difficulties Questionnaire; SE, standard error; SH, self‐harm; SI, suicidal ideation.

There was minimal evidence for multicollinearity in all models, with the largest variance inflation factor (VIF) observed for PHQ‐9 depression scores (VIF = 3.31) and all other VIF <3.

## Discussion

This study aimed to identify mental health, social‐, lifestyle and school‐level factors associated with self‐harm, suicidal ideation and suicide attempts. Close to 1 in 5 adolescents reported lifetime self‐harming behaviour at baseline and 12‐month follow‐up, which is in line with previous research (Lim et al., 2019). The proportion of participants reporting suicidal ideation in the past month at baseline (23.9%) and follow‐up (18.6%) was higher than the 12‐month prevalence rates reported in a recent global review of adolescent suicidal behaviour (Van Meter et al., 2023). This difference may reflect the variable time frames assessed or regional variations in suicide, with the rates obtained in the current study similar to the total rate reported for studies in Australia and New Zealand (18.9%) (Van Meter et al., 2023). Suicide attempt rates in the past 12 months were lower in the current study than reported in the previous review (Van Meter et al., 2023), and this may reflect the younger age of participants in the current study, with suicidal ideation and attempts tending to increase rapidly with age during adolescence (Nock et al., 2013).

Not surprisingly, a previous mental health diagnosis or history of self‐harm, suicidal ideation and/or suicide attempt reported at baseline was significantly associated with most outcomes at 12‐month follow‐up (Richardson et al., 2024; Valencia‐Agudo et al., 2018). Once these factors were accounted for, several other predictors of self‐harm, suicidal ideation and suicide attempt were also identified. For instance, female and other gender identities, as well as LGBTQA+ identity, were found to be significantly associated with self‐harm and suicidal ideation, reflecting previous review findings (Richardson et al., 2024; Surace et al., 2021). No association was found in the current study between gender, LGBTQA+ status and suicide attempts. This may reflect the low suicide attempt base rate in the current study, as these factors have tended to also be associated with suicide attempts in the literature (Richardson et al., 2024; Surace et al., 2021).

Similarly to previous research, higher levels of peer problems were associated with suicidal ideation and suicide attempts in the current study (De Luca et al., 2022; Evans et al., 2004). Being cyber‐bullied was also found to be associated with suicidal ideation (Richardson et al., 2024) and was a significant predictor of suicide attempts before controlling for baseline self‐harm and suicidality. These findings suggest that peer problems (including cyberbullying) may be a particular risk factor for suicidal ideation and attempts and differentiate them from self‐harm. In line with previous research (Richardson et al., 2024; Wasserman et al., 2021), the current study also found the association between bullying and suicidal ideation and attempts to be stronger for cyberbullying compared with face‐to‐face bullying.

A positive association between higher prosocial behaviour and self‐harm and suicidal ideation was also identified, which was somewhat unexpected (Taliaferro, Heerde, Bailey, Toumbourou, & McMorris, 2023; Yang, Kim, Kim, & Hwang, 2022), with the drivers of this association not entirely clear. Given the number of independent variables in the model, it is possible that complex interactions with other indicators led to a finding based on residuals rather than a true effect, particularly as univariate associations were not significant. It may also be the case that prosocial young people are more vulnerable to peer comments and disruptions in social connections that are common in this age group (Law & Shek, 2016; Ma, Batterham, Calear, & Han, 2019). Future qualitative and quantitative research may assist in further elucidating this finding. Depressive symptoms were significantly associated with suicidal ideation but not the other outcome measures, while none of the other symptom measures were found to be significantly associated with self‐harm, suicidal ideation or suicide attempts. This may reflect complex interactions between independent variables having an influence on findings.

None of the protective factors assessed in the current study, such as family social support, school connectedness or school climate, were identified as significant predictors of self‐harm, suicidal ideation or suicide attempts in the final models. This was surprising given their previous cross‐sectional and prospective associations with lower self‐harm and suicide risk in the literature (Evans et al., 2004; Marraccini & Brier, 2017; Richardson et al., 2024; Valencia‐Agudo et al., 2018; Wasserman et al., 2021). The lack of positive associations in the current study could reflect potential interaction effects between variables, particularly given that positive family interactions were significantly associated with lower levels of self‐harm and suicidal ideation in initial models, before baseline levels of self‐harm and suicidal ideation were accounted for. Based on the strength of previous findings, further investigation of these protective factors is warranted.

Given recent commentary about social media driving rises in self‐harm and suicide risk in young people (Christensen, Slade, & Whitton, 2024), it is interesting to note that no such associations were identified in the current study. Greater recreational screen use was found to be associated with lower suicidal ideation, while maladaptive social media use was not associated with self‐harm, suicidal ideation or suicide attempts. Given these findings, and the converse findings for cyberbullying, more research is needed to better understand the conditions under which aspects of social media and recreational screen use can be detrimental versus protective to young people's mental health. Additionally, there would be value in elucidating for whom social media has negative and positive effects (Davis & Goldfield, 2025) and identifying the underlying mechanisms that could explain the association between social media use and poorer mental health in some young people (Orben, Meier, Dalgleish, & Blakemore, 2024).

Collectively, the risk factors identified in the current study are reflective of those identified in a recent umbrella review (Richardson et al., 2024) and further highlight the need to target these in prevention and early intervention programs. For example, there may be value in delivering educational and therapeutic interventions in schools that can better equip young people to manage their mental health and peer relationships or improve their digital literacy skills to improve online safety and reduce cyberbullying. Equipping parents with the knowledge and skills to better support their child's mental health may also be beneficial and is a notion supported by a recent review of common elements in treatments for youth suicide attempts and self‐harm that identified the provision of therapy and skills training for youth and family/caregivers as important (Meza, Zullo, Vargas, Ougrin, & Asarnow, 2023). The risk factors identified in the current study also have the potential to identify groups in need of further screening or targeted support for self‐harm and suicide risk. This may be particularly relevant to the vulnerable populations identified in the current study, such as females and young people reporting diverse gender and sexual identities.

There are a number of clear strengths associated with the current study, including the large and diverse sample, longitudinal research design and the broad range of mental health, social and lifestyle factors assessed, as well as self‐harm, suicidal ideation and suicide attempts. Alongside these strengths, though, are some limitations. All of the factors and outcomes in the current study were assessed using self‐report measures and thus are open to reporting biases. Rates of self‐harm, suicidal ideation and suicide attempts were lower at 12 months than at baseline. This may reflect some participants electing not to disclose repeat self‐harm, suicidal ideation or attempts at 12 months due to reluctance in being followed up in accordance with study risk protocols or due to a range of other known reporting biases (Klimes‐Dougan, Mirza, Babkin, & Lanning, 2022).

Although suicidal ideation and suicide attempts were measured prospectively, covering the 12 months from baseline to follow‐up, self‐harm was based on lifetime reports, providing weaker evidence for the direction of observed associations. Interpretation of self‐harm findings therefore should consider that the self‐harm outcome may have included episodes from before the follow‐up period. Longitudinal examination of additional characteristics associated with suicidal behaviour, such as hospitalisations and severity of self‐harm episodes, may provide further insights into optimal approaches for adolescent suicide prevention. Some of the measures used in the current study, such as screen and social media use, may not have adequately captured the harmful (or protective) aspects of the factors assessed, while some of the SDQ subscales had suboptimal internal consistency and thus results should be viewed with this in mind.

While the baseline sample was broadly representative of the general Australian population with respect to gender, remoteness and other diversity characteristics, it was marginally more advantaged and less linguistically diverse (Werner‐Seidler et al., 2023) and did not include young people not in formal schooling. The sample at the 12‐month assessment was healthier than the baseline sample due to differential attrition, with participants with suicidal ideation marginally less likely to complete this assessment (26% attrition for those with SI vs. 23% for those with no SI). Effects of mental health on attrition were significant, although with small or very small effect sizes. Augmenting the findings with data from clinical samples may be useful for more fully characterising the observed relationships.

## Conclusion

Overall, the results of the current study highlight that young people who identify as female, gender diverse, LGBTQA+ and report a previous mental health diagnosis may be at greatest risk of self‐harm, suicidal ideation and suicide attempt. Depression, peer problems and online bullying may be good targets for future prevention and early intervention programs, and family support may be a protective factor that could be promoted to reduce risk. With data for this cohort study continuing to be collected, changes in risk and protective factors over time will be able to be assessed, as well as the identification of common trajectories of self‐harm and suicide risk across adolescence and the factors associated with them. Collectively, the outcomes of this research will assist in better understanding self‐harm, suicidal ideation and suicide attempts in adolescents and improving prevention and screening approaches.

## Ethical information

Parents/guardians and students provided active informed consent and assent, respectively, for students to participate in the study. The Future Proofing Study received ethics approval in 2019 from the University of New South Wales Human Research Ethics Committee (HC180836), the NSW Government State Education Research Applications Process Approval (SERAP 2019201) and relevant Catholic Schools Dioceses across Australia.

**Key points**

- Rates of self‐harm and suicide are high in young people, and it is important to identify risk and protective factors associated with these outcomes to enable early identification and intervention.
- The current study overcomes the limitations of previous research by assessing, longitudinally, the effect of a range of mental health, social, lifestyle and school‐level factors on both self‐harm and suicide outcomes.
- Young people who identify as female, gender diverse, LGBTQA+ and report a previous mental health diagnosis may be at greatest risk of self‐harm, suicidal ideation and suicide attempt.
- Depression, peer problems and online bullying may be good targets for future prevention and early intervention programs, and family support may be a protective factor that could be promoted to reduce risk.

## Data Availability

The data that support the findings of this study are available from the corresponding author upon reasonable request. The data are not publicly available due to privacy or ethical restrictions.
